# Arginine vasopressin V1a agonist attenuates methicillin-resistant *Staphylococcus aureus*-induced vascular leakage by inhibiting bradykinin

**DOI:** 10.1186/cc12997

**Published:** 2013-11-05

**Authors:** Perenlei Enkhbaatar, Sebastian Rehberg, Osamu Fujiwara, Ernesto Lopez, Donald S Prough

**Affiliations:** 1 Department of Anesthesiology, University of Texas Medical Branch, Galveston, TX, USA

## Background

Previously, we have shown that methicillin-resistant *Staphylococcus aureus *(MRSA) sepsis was associated with more severely pronounced vascular leakage compared with *Pseudomonas aeruginosa *sepsis. We have also demonstrated that the arginine vasopressin V1a receptor (V1aR) agonist significantly attenuated the severity of MRSA-induced vascular leakage [1]. The goal of the present study was to explore mechanistic aspects of V1aR agonist's action.

## Materials and methods

Twelve adult female sheep were operatively prepared for chronic study. After 5 days of recovery, tracheostomy was performed under anesthesia and injury was given. The injury consisted of insufflation of cooled cotton smoke (48 breaths) and instillation of 2.5 × 10^6 ^CFU MRSA into the lungs by bronchoscope under maintenance isoflurane anesthesia. Following the injury, sheep were awakened, placed on mechanical ventilation and randomly allocated into two groups: control group, saline treated, *n *= 6; and POV group, treated with intravenous V1aR agonist, Phe2-Orn8-Vasotocin (POV) (Ferring Research Institute, Inc., San Diego, CA, USA), *n *= 6. The titration of POV was started when mean arterial blood pressure (MAP) dropped by 10 mmHg from the baseline with the initial dose of 30 pmol/minute, which was further adjusted to maintain MAP close to baseline. All sheep were resuscitated with lactated Ringer's solution with initial rate of 2 ml/kg/hour that was further adjusted according to hematocrit. The experiment lasted 24 hours. Plasma levels of nitric oxide (NO; Grease reaction), asymmetric dymethylarginine (ADMA; mass spectrometry) and bradykinin (mass spectrometry) were determined at 0 hours and every 3 hours after the injury.

## Results

MRSA-induced plasma levels of NO (nitrite/nitrate) as well as cumulative body fluid were significantly inhibited by V1aR agonist. The treatment with POV also attenuated the MRSA-induced hypotension. The plasma levels of ADMA were higher in the treated group compared with the control at 24 hours after the injury (0.93 ± 0.14 in control, *n *= 3 vs. 1.23 ± 0.08 in POV, *n *= 6). In addition, the treatment with POV significantly inhibited the MRSA-induced bradykinin increases at 3 hours after the injury (1.14 ± 0.4 in control vs. 0.52 ± 0.001 in POV, *P *< 0.05).

## Conclusions

Arginine vasopressin V1aR agonist attenuates the severity of MRSA-induced vascular leakage by inhibiting potent permeability factor bradykinin and excessive NO. The V1aR agonist may modulate NO production by promoting the release of endogenous NO synthase inhibitor ADMA.
